# Abstracts from Foreign Journals

**Published:** 1902-11

**Authors:** S. J. J. Harger

**Affiliations:** Philadelphia, Pa.


### ABSTRACTS FROM FOREIGN JOURNALS.

### FRENCH.

Under the Direction of S. J. J. Harger, V.M.D.,
PHILADELPHIA, PA.

Treatment of Corneal Ulcers. Ulcerations of the cornea as a complication of simple keratitis, distemper, and malnutrition are common. Cadiot recommends the instillation of a solution of permanganate of potash, 1 : 200. He cites some cases in which by this treatment he has obtained rapid recoveries.—*Rev. Vet.*

Castration of the Bitch. To produce anæsthesia Ehrhardt gives subcutaneously $\frac{3}{4}$ to $1\frac{1}{2}$ grains of morphine, according to the size of the animal. A ligature is placed around the ovarian ligaments. The ovary is then severed from its attachment with scissors, and the broad ligaments cut to the bifurcation of the horns. The same is repeated on the opposite side, the uterus ligatured, and the ovaries and cornu removed.

He practised simple ligation of the ovarian ligaments without removing the ovary. Some of these bitches came in heat, and several bore a litter of puppies. Several were recastrated four or five months after the first operation. The ovaries were considerably hypertrophied; in one case there were adhesions with the intestines; the uterine horns were double their volume. In some the ligature was still found, but was more or less encysted.

Ligaturing, therefore, is sufficient to make the bitch infecund. Removal of the uterine horns guards against impregnation if, by accident, a fragment of the ovaries is left in place.—*Ann. de Med. Vet.*

Iodide of Potash in the Treatment of Lameness. The case cited by Schenkel was a horse lame for three weeks. Although there was no exostosis, spavin was diagnosed, and a blister applied. After eighteen days of rest the lameness was as marked as before. Hip-joint lameness was then suspected, and liniments were applied upon this region.

After eight weeks there was no sensible improvement. Supposing that there might be a specific, probably rheumatismal arthritis, 10 grains of iodide of potash were administered daily in the feed. In five days the lameness was much improved, and in five days more it had completely disappeared.

Schenkel recalls lameness in the foreleg of a dog as a sequel to distemper. Stimulating applications were ineffective, and the lameness disappeared completely after the daily administration of 1 grain of iodide of potash.—*Ann. de Med. Vet.*

Multiple Cerebral Hemorrhages in the Horse. A horse, seven years old, fat and plethoric and in good spirits, had taken active exercise on a trot and gallop. The next morning he was immobile and unable to get up; when raised he tottered, and could stand only with difficulty; respirations regular; pulse full and rapid; temperature 39.6° in the morning and 41.1° in the evening.

The treatment consisted in bleeding—four quarts—stimulative, and laxative preparations. Prostration and coma increased, and, finally, all functions ceased.

At the autopsy the central nervous system in general was of a citrine color, especially around the hemorrhagic areas, due to rapid decomposition of the blood. Abundant multiple hemorrhages were found in the cerebellum; in the cerebral hemispheres they were quite extensive. The corpora striata, cerebral peduncles, and the medulla were intact, which explains the slow evolution of the symptoms.—*Ann. de Med. Vet.*

Fistula of the Poll from Foreign Body. An Anglo-Norman mare running in the field produced a contused wound of the poll, which afterward suppurated. This wound cicatrized rapidly under the ordinary surgical treatment. One morning the coachman while grooming the mare found a small fistula, from which a few drops of pus were discharged, and necrosis of the nuchal ligament was suspected.

At the Lyons Veterinary School Cadéac opened the fistula and found several pieces of wood, forming a combined mass of one cubic centimetre, encysted, and resting upon the occipital bone. The bone was curetted and the tract cleansed with corrosive sublimate; cicatrization followed rapidly.—*Ann. Vet. Med.*

Meningitis in the Dog. Meningitis may result from distemper, traumatisms and injuries, and diseases of the surrounding tissues. The symptoms are usually attributed to cerebral troubles.

Among the most common causes of cerebral meningitis are injuries to the eyes and affections of the ears. Cadéac mentions a bull-dog with the eye perforated by a cat; no treatment was at first given, and the eye soon suppurated, involving the deeper tissues. Two days afterward symptoms of meningitis, accompanied by vomition, developed. The animal succumbed suddenly, and the necropsy revealed a fibrino-purulent exudate into the meninges, especially at the base of the brain.

Tumors of the ocular globe and of the sinuses sometimes cause meningitis by causing absorption of the cranial wall. Traumatisms of the cranial wall, on the other hand, produce concussion and injure the cerebrum without determining any meningeal troubles. Trephanation of the cranium, so dangerous in the horse, is benign in the dog.

Meningitis of auricular origin is not rare. It may succeed chronic auricular catarrh, tumors, and infection of the middle ear due to alterations in the Eustachian tube. Cadéac mentions a dog, two years old, succumbing in three days to cerebro-spinal meningitis consecutive to an internal otitis resulting from an infection propagated successively by continuity of structure from the trumpet of the Eustachian tube to the external ear, and from the latter through the auditory nerve to the meninges and the cerebrum.—*Ann. de Med. Vet.*

Generalized Alopecia in a Cow. Vaché reported a cow, nine years old, which had at the age of five years circular bald patches on the skin which increased and finally coalesced until, at the end of a year, the entire body was denuded of its hair excepting a few on the left cheek and the end of the tail. All applications were useless. The skin was of a brilliant black color. The animal was otherwise in perfect health.

Hæmoglobinuria of Muscular Origin. The pathogenesis of hæmoglobinuria as seen in so-called azoturia is in many points still obscure. Classical writers claim that hæmoglobinuria results from the red blood corpuscles. Some assert that this destruction takes place in the blood—hæmoglobinæmia—and others, that the kidneys are the seat of the disorganization—renal theory.

Camus and Pagniez have endeavored to determine if hæmoglobin can be found in other parts of the body, and confined their studies to the striated muscle tissue. They have prepared a muscle juice as follows: From a chloralized dog 15 grammes of muscle were removed, triturated with enough cold distilled water to obtain a filtrate of 50 to 60 c.c., to which was added enough sodium chloride to make it isotonic with the blood.

This solution is then introduced into the saphena vein of an anæsthetized dog. In from ten to thirty minutes the urine removed with a catheter is rose-colored, and the spectroscope shows the two rays of oxyhæmoglobin; there is hæmoglobinuria. There are no blood corpuscles in the urine. They have found that in order to produce hæmoglobinuria by injecting hæmoglobin obtained from the blood corpuscles a larger quantity of the latter is required.

The authors then experimented to determine if this phenomenon could result in consequence of muscular lesions. They injected 150 grammes of water at 5° C. into the thigh muscles of a chloralized dog; trembling and contraction followed. In half a minute hæmoglobinuria was evident.

Then arose the question whether the muscle juice does not contain some substance which may cause the elimination through the kidney of the hæmoglobin of the blood. The muscle juice was heated for fifteen minutes at a temperature of 56°, and when injected alone after cooling or mixed with hæmoglobin no hæmoglobinuria followed. It will thus be seen that muscle juice contains a substance that acts upon the kidneys, producing hæmoglobinuria, and that the muscle hæmoglobin passes through the kidneys more readily than that obtained from the blood cells.

These results seem to sustain the theory that *hæmoglobinuria a frigora* (azoturia) is at present a breaking down of the muscles, possibly from a myositis rather than a destruction of the red blood cells. According to Jobelot, myositis always exists in this affection. This is the theory entertained by most German authors.—*Ann. de Med. Vet.*

---

Dr. D. B. Dennish, of Trenton, N. J., was a visitor to Philadelphia in October.

